# Gene editing technology to improve antitumor T-cell functions in adoptive immunotherapy

**DOI:** 10.1186/s41232-024-00324-7

**Published:** 2024-03-11

**Authors:** Yusuke Ito, Satoshi Inoue, Yuki Kagoya

**Affiliations:** Division of Tumor Immunology, Institute for Advanced Medical Research, Keio University School of Medicine, Tokyo, 160-8582 Japan

**Keywords:** Adoptive immunotherapy, Chimeric antigen receptor, Epigenetics, DNA methylation, PRDM1, Memory T cell, T-cell exhaustion, CRISPR/Cas9

## Abstract

Adoptive immunotherapy, in which tumor-reactive T cells are prepared in vitro for adoptive transfer to the patient, can induce an objective clinical response in specific types of cancer. In particular, chimeric antigen receptor (CAR)-redirected T-cell therapy has shown robust responses in hematologic malignancies. However, its efficacy against most of the other tumors is still insufficient, which remains an unmet medical need. Accumulating evidence suggests that modifying specific genes can enhance antitumor T-cell properties. Epigenetic factors have been particularly implicated in the remodeling of T-cell functions, including changes to dysfunctional states such as terminal differentiation and exhaustion. Genetic ablation of key epigenetic molecules prevents the dysfunctional reprogramming of T cells and preserves their functional properties.

Clustered, regularly interspaced, short palindromic repeats (CRISPR)/CRISPR-associated protein (Cas)-based gene editing is a valuable tool to enable efficient and specific gene editing in cultured T cells. A number of studies have already identified promising targets to improve the therapeutic efficacy of CAR-T cells using genome-wide or focused CRISPR screening. In this review, we will present recent representative findings on molecular insights into T-cell dysfunction and how genetic modification contributes to overcoming it. We will also discuss several technical advances to achieve efficient gene modification using the CRISPR and other novel platforms.

## Introduction

Among a number of cancer immunotherapies, adoptive immunotherapy is a unique approach in that it uses antitumor immune cells as a living drug. Beginning with pioneering studies using tumor-infiltrating lymphocytes [1], genetically engineered T-cell therapy has emerged as a robust approach to rationally generate antitumor T cells against defined target antigens [2].

In addition to using the T-cell receptor (TCR), T cells can be engineered with a chimeric antigen receptor (CAR), a synthetic receptor that enables T cells to recognize and eliminate target cells expressing the cognate ligand irrespective of the HLA type [3]. Adoptive transfer of CAR-T cells is an approach now used to treat several types of blood cancer in the clinic. Tremendous clinical outcomes have been achieved for patients with B-cell lymphoma and acute lymphoblastic leukemia (ALL) who received autologous CD19-targeted CAR-T cells [4–8]. However, even with a remarkable initial response, relapse and refractory cases after treatment with CAR-T cells are frequently observed [8]. Furthermore, no equivalent clinical success has been achieved to date for most solid tumors or even for the majority of hematologic malignancies [9–11]. These disappointing results can be attributed to several factors: the lack of suitable tumor-specific antigens, insufficient trafficking of infused CAR-T cells into the tumor site, immunosuppressive tumor microenvironment, and the quality of the infused CAR-T cells (capacity for long-term survival and cytotoxicity).

Many investigators are currently addressing these obstacles by using a variety of genetic engineering strategies to enhance CAR-T-cell efficacy and overcome treatment resistance. Not limited to the traditional approach of ectopic transgene expression, T cells can now be engineered with a specific region of the genome to knock out genes or to knock in sequences of interest. By targeting genes that are centrally involved in the induction of T-cell dysfunction, CAR-T cells can achieve enhanced and durable antitumor efficacy. Epigenetic factors are particularly important targets because their manipulation globally remodels T-cell properties [12]. In this review, we provide an overview of current strategies in development to improve the properties of CAR-T cells.

### Nature of epigenetics

Epigenetic regulation is broadly defined as the means by which identical genotypes (DNA sequences) give rise to disparate phenotypes. Epigenetic factors are responsible, at least in part, for how immune cells differentiate, adapt to changes in the microenvironment, and propagate cellular state by controlling chromatin structure and gene expression patterns. This regulation mainly takes the form of DNA modifications such as cytosine methylation or hydroxy methylation and histone modifications such as amino acid acetylation or methylation [13].

DNA methylation occurs when a methyl group is attached to the cytosine component of a cytosine-guanine dinucleotide (CpG) in a reaction catalyzed by DNA methyltransferases (DNMTs). The cytosine in the methylated CpG can be oxidized by the methylcytosine dioxygenase ten-eleven translocation (TET) enzymes to yield 5-hydroxymethyl cytosine (5-hmC), which can be converted back into unmodified cytosine via multiple mechanisms [14]. Such methylation of CpG islands near transcription start sites or proximal promoter loci results in suppression of gene transcription, either by directly abrogating the capacity of the DNA to permit the binding of transcription factors or by recruiting histone-modifying enzymes that further block transcription as described below.

The second type of epigenetic regulation involves posttranslational modifications of histone proteins, mainly in the form of methylation or acetylation of specific amino acids [15]. Histone methyltransferases add methyl groups to lysine residues in the tails of histone proteins, particularly histones H3 and H4. For example, trimethylation of lysine 9 or lysine 27 of histone H3 (H3K9me3/H3K27me3), or lysine 20 of histone 4 (H4K20me3), is prevalent in heterochromatin and mediates transcriptional suppression. In contrast, methylated H3K4, H3K36, and H3K79 are present in euchromatin and correlate with active transcription. Methyl groups in a histone are removed by histone demethylases such as LSD1 (encoded by *KDM1A*) and KDM6A/B (encoded by *UTX* and *JMJD3*), and the balance of histone methyltransferases versus demethylases creates a dynamic remodeling of chromatin. Similarly, histone acetyltransferases are epigenetic enzymes that transfer acetyl groups to lysine residues, which is associated with transcriptional activation. Histone deacetylases (HDAC) remove acetyl groups from histone lysine residues, leading to increased chromatin condensation and the silencing of gene transcription.

### CAR-T-cell persistence

The adaptive immune response allows an organism that has previously been exposed to a foreign antigen to quickly recognize and eliminate that antigen if it reappears. Naive T cells that recognize their cognate antigens undergo clonal expansion and differentiate into effector T cells and then into long-lived memory T cells. A sustained antitumor immune response requires that tumor-reactive memory T cells persist and function upon repeated antigen exposure. However, most infused CAR-T cells disappear rapidly after initial expansion, allowing residual tumor cells to regrow [16, 17]. These limitations warrant the search for factors that can be modified to prolong the lifespan of CAR-T cells.

Memory T cells are phenotypically and functionally divided into stem cell-like memory (T_SCM_, CD45RA^+^CCR7^+^CD62L^+^CD27^+^CD28^+^IL-7R^+^CD95^+^), central memory (T_CM_, CD45RA^−^CCR7^+^CD62L^+^CD27^+^CD28^+^IL-7R^+^CD95^+^), and effector memory (T_EM_, CD45RA^−^CCR7^−^CD62L^−^CD27^+/-^CD28^+/-^IL-7R^+/-^CD95^+^) T cells [18]. In preclinical models, CAR-T cells with a T_SCM_ or T_CM_ phenotype (which are less differentiated) outperformed more differentiated counterparts [19–22]. The memory differentiation status of infused CAR-T cells was also associated with clinical outcomes [20, 23, 24]. These observations highlight the importance of the differentiation status of infused CAR-T cells and the desirability of establishing protocols leading to cell products with less differentiated, stem cell-like characteristics [25].

### Epigenetic regulation of T-cell differentiation

**Fig. 1:**
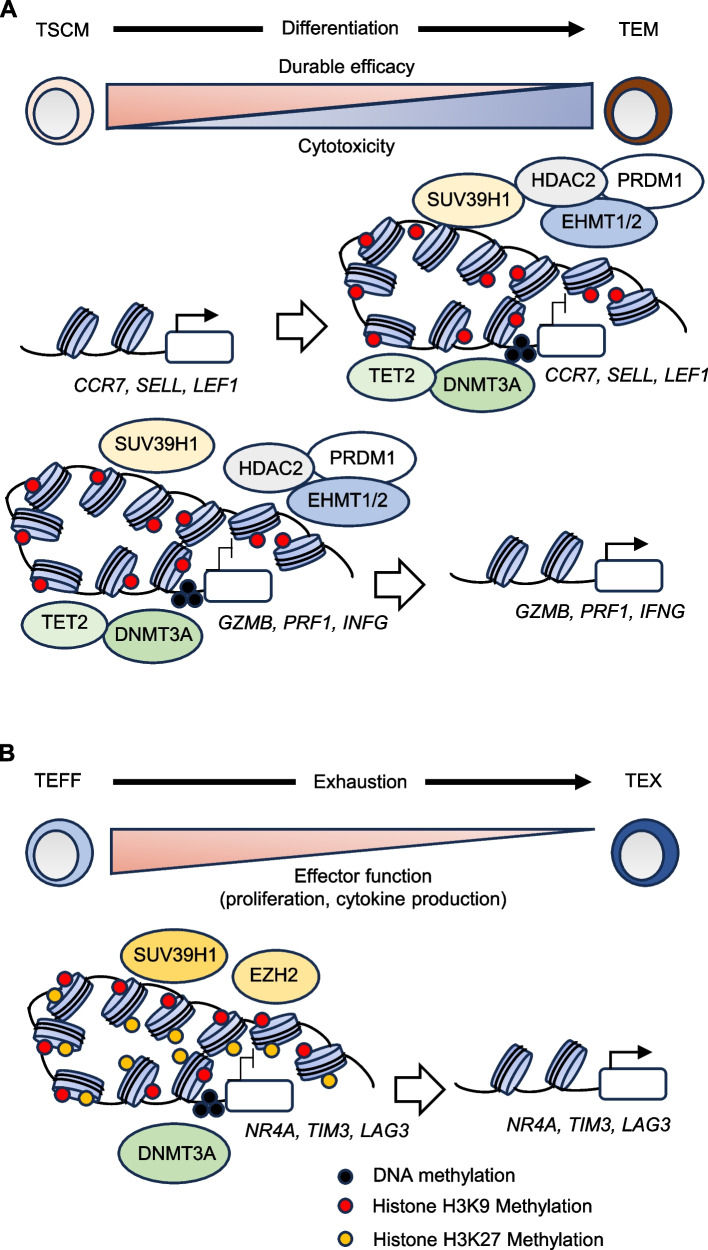
Epigenetic changes associated with CAR-T-cell differentiation and exhaustion. Epigenetic profiles such as DNA or histone H3K9/K27 methylations are altered during **A** differentiation of stem cell-like memory T (T_SCM_) cells into effector memory T (T_EM_) cells or **B** T-cell exhaustion. **A** Transcriptional repression by DNA or histone modification at the promoter/enhancer regions of native T-cell-associated genes (*TCF7*, *SELL*, *CCR7*, and *LEF1*) by epigenetics factors including DNMT3A, TET2, SUV39H1, and PRDM1. **B** Expression of exhaustion-associated genes (*NR4A1/2/3*, *HAVCR2*, *TOX*, and *LAG3*) is also regulated by epigenetic mechanisms such as DNA methylation or histone H3K9/K27 methylation

During the differentiation of naive T cells into T_SCM_, T_CM_, and T_EM_ populations, epigenetic changes occur through DNA methylation or histone modifications due to the orchestrated functions of a variety of transcription factors (Fig. 1A). In the case of DNA methylation, DNMT3A and TET2 play a critical role in T-cell fate decisions. Long-lived memory cells can arise from a subset of effector T cells through de-differentiation induced by epigenetic mechanisms: promoter regions of naive T-cell-associated genes, such as *SELL*, *CCR7*, and *TCF7*, undergo demethylation upon memory formation [26]. Memory formation was promoted by genetic ablation of DNMT3A through increased DNA demethylation of naive-associated genes. Interestingly, *Tet2* knockout mice also showed increased memory formation, which appeared to be mediated by increased DNA methylation at several key effector-associated transcription factors, including T-box transcription factor 21 (*Tbx21*, encoding T-bet) and PR domain zinc finger protein 1 (*Prdm1*) [27]. Similarly, in adoptive immunotherapy, DNA methylation profiling of the infused CAR-T cells revealed genome-wide DNA methylation changes during therapy [28]. These changes included the repression of stem-associated genes and the upregulation of effector-related genes, correlating with a lack of efficacy in these patients.

With respect to histone modifications in this context, less-differentiated cells such as T_SCM_ cells showed enrichment in activating histone marks (H3K4me3) and depletion of repressive marks (H3K27me3) in the promoter regions of genes linked to immunological memory (*TCF7*, *LEF1*, *FOXO1*) [29]. These less-differentiated cells also featured repressive histone marks (H3K27me3) in the promoters of effector-related genes (*IFNG*, *TBX21*, GZMB, *PRF1*). In the more-differentiated T_CM_ and T_EM_ subsets, the same loci showed progressively fewer activating histone marks and more repressive marks.

Several other studies have demonstrated results consistent with the above observations: (1) Genetical knockout of the histone H3K9 methyltransferase SUV39H1 enhanced the stem cell/memory gene expression program and silenced the naïve T-cell differentiation into effector CD8^+^ T cells in mice after *Listeria monocytogenes* infection [30], and (2) the H3K9 methyltransferase G9a/EHMT and HDAC2 were recruited by *PRDM1* to the *CD27* and *IL2RA* promoters, which accelerated effector T-cell differentiation [31, 32].

### Reprogramming of epigenetics to improve CAR-T-cell persistence

The above studies strongly suggest that reproducing epigenetic patterns that favor memory over effector T-cell differentiation may be a promising strategy to promote CAR-T-cell longevity. A striking in vivo demonstration of the power of disrupting epigenetics to improve CAR-T-cell efficacy has been published [33]. A patient with CLL was treated with CD19-targeted CAR-T cells with biallelic TET2 disruption due to insertion of the CAR gene. The TET2-deficient clone expanded massively in vivo and generated the majority of circulating CAR-T cells, resulting in long-term disease remission. These findings bolstered the concept that epigenetic reprogramming of CAR-T cells by genetic engineering/editing technology could be used to produce long-lived and more efficacious CAR-T cells. Indeed, we and others have reported that pharmacologic or genetic inhibition of *ARID1A*, *DNMT3A*, *TET2, SUV39H1*, and *PRDM1* in CAR-T cells prevents the formation of epigenetic programs associated with terminal T-cell differentiation [33–37]. T_SCM_/T_CM_-like CAR-T cells were significantly maintained after prolonged antigen stimulation. These properties have ultimately translated into enhanced T-cell persistence and superior tumor control.

### Exhaustion of CAR-T cells

T-cell exhaustion is considered to be another major hurdle compromising the efficacy and applicability of CAR-T cell therapy. When an immune response is prolonged, the responding T cells become exhausted, a state of dysfunctionality characterized by poor proliferative capacity, decreased persistence, and diminished effector functions. Exhausted T cells show upregulated expression of inhibitory receptors such as programmed cell death-1 (PD-1), cytotoxic T-lymphocyte antigen-4 (CTLA-4), lymphocyte activation gene-3 (LAG3), and T-cell immunoglobulin domain-3 (TIM3), as well as an impaired ability to produce cytokines such as IFN-γ, TNF-α, and interleukin-2 (IL-2) [38]. These detrimental changes can reduce the effectiveness of T-cell-mediated immune responses against not only chronic viral infections but also cancers [39]. Higher expression of exhaustion markers (PD-1, TIM3, LAG3) by infused CAR-T cells correlated with poor treatment response in CLL patients [23].

In addition to prolonged antigen exposure, antigen-independent CAR signaling induced by self-aggregation of the CAR molecule has been implicated in T-cell exhaustion. This aggregation induces spontaneous T-cell activation, known as tonic signaling, which is associated with reduced antitumor activity of CAR-T cells [40–42]. That being said, a different study showed that tonic signaling in CAR-T cells could result in enhanced in vivo potency and persistence under some circumstances [43]. These contrasting observations indicate that the effect of tonic signaling in CAR-T cells may be highly context dependent and warrants further investigation.

### Epigenetic regulation of T-cell exhaustion

Epigenetic changes are a critical feature of T-cell exhaustion. Exhausted T cells show a consistent epigenetic profile that is distinct from that associated with functional effector and memory T cells (Fig. 1B) [44]. The epigenetic profiles acquired by exhausted T cells were also maintained following the administration of anti-PD-1, anti-PD-L1, or anti-CTLA-4 antibodies as immune checkpoint blockade therapy intended to rejuvenate T cells and increase their survival and proliferation [45]. Genome-wide DNA methylation profiling was performed on antigen-specific mouse CD8^+^ T cells to compare effector and exhaustion phases of an immune response. This work showed that the transition from effector to exhausted T cells is mediated by DNMT3A [46]. Recently, genome-wide CRISPR screens in both mouse and human tumor models have revealed ablation of cBAF (canonical BRG1/BRM-associated factor) family members such as ARID1A counteracts the development of T-cell exhaustion [47].

Several groups have performed epigenetic remodeling interventions to increase epigenetic plasticity and reverse the exhaustion of T cells that occurs during immunotherapy. For example, genetic ablation or pharmacologic inhibition of MAP4K1 prevents T-cell exhaustion via the downregulation of the NFκB-PRDM1 cascade and improves CAR-T-cell efficacy [48]. DNMT3A- or SUV39H1-deficient CAR-T cells epigenetically downregulated exhaustion genes, resulting in enhanced antitumor efficacy in preclinical mouse models [35, 36]. Another team showed that during T-cell exhaustion, epigenetic remodeling promoted the recruitment of NR4A family members to the promoter/enhancer regions of the *PDCD1* and *TOX* genes [49]. Indeed, genetic knockout of NR4A in CAR-T cells resulted in epigenetic reprogramming that reduced exhaustion and promoted their antitumor activity [49]. Although several epigenetic factors are overlappingly associated with both terminal T-cell differentiation and exhaustion, these phenomena are not necessarily induced at the same time. Recent studies have shown that a subset of exhausted T cells possess share phenotypic characteristics with early memory T cells and have been termed precursor exhausted T cells [50]. Indeed, we have reported that while PRDM1 KO CAR-T cells showed improved persistence and proliferation compared to conventional CAR-T cells, they still exhibited *TOX* upregulation and PD1 elevation [37]. The antitumor efficacy of *PRDM1* KO CAR-T cells could be further enhanced by concomitant knockout of *NR4A3* by repressing the exhaustion epigenetic program [51].

In addition to the epigenetic modification, antitumor functions of CAR-T cells can be potentiated through ablation of various genes. For example, *PDCD1* [52, 53], *CTLA4* [54], *TGFBRII* [55], and *A2AR* knockout [56] can block the suppressive signals from the tumor microenvironment and enhance effector functions of CAR-T cells.

### CRISPR/Cas9-mediated gene editing

**Fig. 2:**
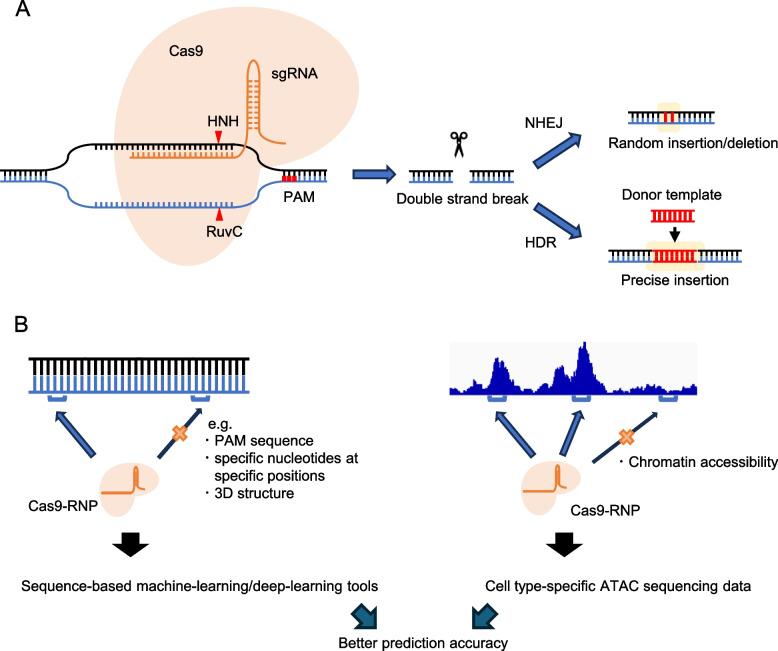
CRISPR/Cas9-mediated gene editing. **A** CRISPR/Cas9 technology can knock out specific genes through DNA double-strand breaks followed by nonhomologous end joining (NHEJ). It can also insert donor oligo by the mechanism of homology-directed repair (HDR). **B** Accurate selection of genomic sequences targeted by guide RNAs can be improved by combinatorial use of sequence-based prediction and reference to epigenetic profiles at the target site

As shown in the examples above, ablation of specific genes is a key modification to enhance CAR-T cell properties. Clustered, regularly interspaced, short palindromic repeats (CRISPR)/CRISPR-associated protein (Cas) technology has revolutionized the field of gene editing, surpassing previous technologies such as zinc finger nucleases (ZFNs) and transcription activator-like effector nucleases (TALENs) [57, 58]. The CRISPR/Cas system consists of Cas nuclease and guide RNA (gRNA) [59]. Several Cas nucleases have been used for CRISPR/Cas-mediated gene editing, including *Staphylococcus pyogenes* Cas9 (SpCas9), *Staphylococcus aureus* Cas9 (SaCas9) [60], and *Acidaminococcus* sp. Cas12a (AsCas12a, also known as Cpf1) [61]. Among them, SpCas9 is the most widely used due to its high efficiency in gene editing and simple protospacer adjacent motif (PAM) sequence (5`-NGG-3`) (Fig. 2A) [62]. The gRNA for SpCas9 is comprised of a CRISPR RNA (crRNA) with a 20-nucleotide (20-nt) sequence recognizing target DNA sites and a trans-activating CRISPR RNA (tracrRNA), which provides a scaffold for binding to the Cas9 nuclease. The Cas9 recognizes PAMs located downstream of the target site and causes a double-strand break between positions 17 and 18 of the 20-nt gRNA sequence. The SpCas9 nuclease has two nuclease domains, HNH and RuvC, which cleave the target and nontarget strands, respectively, causing double-stranded DNA breaks (DSBs) [59]. DSBs can be repaired through two pathways, nonhomologous end joining (NHEJ) and homology-directed repair (HDR). NHEJ is an error prone repair pathway and introduces insertions and deletions (indels) around the breakpoint, resulting in the disruption of gene expression (knockout) [63]. HDR precisely introduces desired modifications within the breakpoint in the presence of DNA donor templates (knock-in) [64]. The establishment of these basic CRISPR/Cas9 systems has enabled efficient gene editing of antitumor T cells during in vitro preparation.

### Epigenetic profiles as a guide for prediction of optimal gRNA targets

CRISPR/Cas9 is usually introduced into T cells by transient transfection of a Cas9/gRNA ribonucleoprotein complex. The most important factor for efficient gene editing in this procedure is the selection of gRNA targets (Fig. 2B). To find optimal gRNAs that maximize on-target efficacy while minimizing off-target effects, multiple gRNA design tools have been developed [65–67]. Although the accumulating knowledge of general rules associated with gene editing efficiency has led to the development of hypothesis-driven tools, the gene editing process is highly complex and influenced by various parameters, many of which are still unknown. To overcome this problem, machine learning-based tools have also been developed [68].

Importantly, the efficiency of gene editing varies between cell types, even when using the same gRNAs, suggesting that some cell intrinsic characteristics affect editing efficiency [68]. Epigenetic architecture has been identified as one of those factors, as heterochromatin states impede Cas9 binding to the target region and inhibit DNA cleavage [69, 70]. We have recently shown that the chromatin accessibility of T cells, as measured by ATAC sequencing, supports the selection of gRNAs that allow efficient gene editing in human T cells [71]. The combination of currently available prediction tools with epigenetic information has enabled precise selection of optimal gRNA targets. These technological advances will be instrumental in the efficient exploration of genetic targets to improve antitumor T-cell functions.

### Base editing and prime editing

Leverage the unique properties of Cas9 proteins to recognize specific DNA sequences, and a number of novel systems for gene regulation have been developed. Cas9 with double loss-of-function mutations in the nuclease domains (D10A for the RuvC domain mutation and H840A for the HNH domain mutation), termed dead Cas9 (dCas9), loses DNA nuclease activity but retains the ability to bind target DNA. Fused to a transcriptional regulator, dCas9 can be recruited to promoter regions or transcription start sites of target genes to control target gene expression. Fusion with VP64 and its derivatives upregulates target gene expression (CRISPR activation: CRISPRa) [72]. Conversely, the addition of the Krüppel-associated box (KRAB) transcriptional repressor domain attenuates gene expression (CRISPR interference: CRISPi) [73]. These technologies are useful to comprehensively explore essential regulators associated with antitumor T-cell functions or resistance to T-cell-mediated effector functions in tumor cells [74, 75].

For more precise gene modification, new approaches called base editing and prime editing technologies have been developed [76]. Nickase Cas9 (nCas9), in which either one of the nuclease domains is inactivated (D10A or H840A), can cause single-strand DNA breaks. When nCas9 (D10A) is fused to cytosine deaminase (cytosine base editor), the amine group in cytosine can be removed and replaced with uridine (C:G → U:G) [77]. On the other hand, fusion of nCas9 with deoxyadenosine deaminase derived from TadA in *Escherichia coli* (adenine base editor) results in A:T → C:G [78]. Both base editors can be efficiently introduced into T cells by mRNA electroporation [79]. Allogeneic CD7-targeted CAR-T cells with CD7, CD52, and TCR β chain ablated by base editing are being evaluated for safety in an ongoing clinical study.

Prime editing is another recently developed technology to achieve more versatile gene modifications [80]. In this editing system, nCas9 with the H840A mutation is fused to reverse transcriptase and prime editing guide RNA (pegRNA). The pegRNA consists of gRNA, primer binding site (PBS), and reverse transcriptase template (RTT). gRNA domain in pegRNA binds target sites and recruits Cas9 to make a nick in the nontarget strand via the RuvC nuclease domain. Next, PBS domain in pegRNA binds the cleaved nontarget strand, and RTT domain is reverse-transcribed to DNA, which is integrated into the target sites. Prime editing allows all desired single base substitutions and small indels without double-strand breaks or a donor template, which would be a precious tool if editing efficiency in primary T cells is improved [81, 82].

### Universal CAR-T cells

Current CAR T-cell therapies mostly use autologous T cells as the cell source. In addition to high production costs, the quality of patient-derived T cells is often low due to prior rounds of chemotherapy, potentially compromising the durable efficacy of CAR T cells [83]. To overcome these hurdles, off-the-shelf allogeneic CAR-T-cell therapy has emerged as a promising alternative strategy, in which gene editing technology plays a critical role.

Allogeneic CAR-T-cell infusion is associated with two major problems: (i) graft-versus-host disease (GvHD) and (ii) host-mediated rejection of infused T cells. GvHD is induced by the activation of donor T cells that recognize the recipient tissue through endogenous TCRs. Knockout of either *TRAC* or *TRBC*, which encode TCRα and β chains, respectively, can prevent GvHD without affecting CAR-mediated effector functions [84]. In addition, T cells with CAR integrated into the *TRAC* locus averted tonic signaling and outperformed conventional CAR-T cells in preclinical mouse models [85].

The host immune system attacks HLA-mismatched donor CAR-T cells, leading to graft rejection. The anti-CD52 antibody, alemtuzumab, is used to deplete recipient T cells, providing an advantage to CD52-knockout CAR-T cells [86, 87]. Abrogation of HLA class I and II by targeting *B2M* and *CIITA* can also reduce alloreactivity [88]. Since HLA class I-deficient CAR-T cells are susceptible to attack by NK cells, another strategy such as overexpressing HLA-E on CAR-T cells would be required [89].

### Closing remarks

Genetic interventions during the in vitro manufacturing of CAR-T cells offers the opportunity to maximize CAR-T-cell function and thus clinical efficacy. The goal is to generate long-lived memory-like CAR-T cells without compromising their effector function. Epigenetic modification is one of the strategies to enhance CAR-T-cell persistence, mitigate T-cell exhaustion, and promote trafficking to the tumor. While the evidence on the effects of different gene modifications is accumulating, it would also be important to review individual studies in an integrative and systematic manner.

In addition, it should be noted that epigenome editing may affect multiple pathways in T cells, potentially causing unexpected side effects. As many of the genes targeted by such strategies (*DNMT3A*, *TET2*, *PRDM1*) are also tumor-suppressor genes, loss of these factors could cause an increased risk for T-cell malignancies. The establishment of appropriate and standardized preclinical models is essential to accurately assess the safety of novel genetic manipulations.

## Data Availability

Not applicable.
